# Effect of Adding Red Propolis to Edible Biodegradable Protein Films for Coating Grapes: Shelf Life and Sensory Analysis

**DOI:** 10.3390/polym16070888

**Published:** 2024-03-24

**Authors:** Cristina Tostes Filgueiras, Farayde Matta Fakhouri, Vitor Augusto dos Santos Garcia, José Ignacio Velasco, Gislaine Ferreira Nogueira, Luan Ramos da Silva, Rafael Augustus de Oliveira

**Affiliations:** 1 Faculty of Engineering, Federal University of Grande Dourados (FAEN/UFGD), Dourados 79804-970, MS, Brazil; cristinafilgueiras@ufgd.edu.br (C.T.F.); vitor.as.garcia@unesp.br (V.A.d.S.G.); luanramosea@gmail.com (L.R.d.S.); 2 School of Agricultural Engineering, University of Campinas, Campinas 13083-875, SP, Brazil; augustus@feagri.unicamp.br; 3 Poly2 Group, Department of Materials Science and Engineering, Universitat Politècnica de Catalunya (UPC Barcelona Tech), Carrer de Colom 11, 08222 Terrassa-Barcelona, Spain; 4 Faculty of Agricultural Sciences, São Paulo State University (UNESP), Botucatu 18610-034, SP, Brazil; 5 Department of Biomedical and Health Sciences, Minas Gerais State University, Passos 37900-106, MG, Brazil; gislaine.nogueira@uemg.com.br; 6 Faculty of Food Engineering, University of Campinas, (FEA/UNICAMP), Campinas 13083-970, SP, Brazil

**Keywords:** biodegradable films, edible covering, natural antimicrobial agent, fruit, post-harvest technology, conservation, shelf life, sensory analysis

## Abstract

Red propolis is an active ingredient of great nutritional interest which offers numerous benefits as an antioxidant and antimicrobial agent. Thus, the objective of this research was to evaluate the application of an edible and antimicrobial gelatine coating containing red propolis to increase the shelf life of grapes. Gelatine films with an addition of 5, 10, 15, 20 and 25% of red propolis extract were produced to evaluate their antimicrobial activity using the disk diffusion test in solid media. The films with 25% red propolis extract showed antimicrobial activity against the bacteria *Staphylococcus aureus* and *Pseudomonas aeruginosa*. The grapes were coated with pure gelatine, without a plasticizer and with gelatine with 25% red propolis and then stored for 1, 4, 10, 19 and 25 days at temperatures of 25 °C and 5 °C. The results showed that the gelatine coating with propolis reduced the mass loss of grapes stored at 25 °C for 19 days by 7.82% and by 21.20% for those kept at 5 °C for 25 days. The pH, total titratable acidity, soluble solids and color of the grapes increased due to the ripening process. Furthermore, the sensory acceptability indexes of the refrigerated grapes with coatings were superior (>78%) to those of the control samples (38%), proving the effectiveness of the coatings in maintaining the quality of grapes during storage.

## 1. Introduction

Edible packaging from renewable sources has been widely studied due to the great environmental concern related to the disposal of non-renewable materials from petroleum sources [1]. Packaging that is considered edible can contain, in addition to its traditional composition, macromolecules, plasticizing agents, solvents and pH adjusters and, if necessary, some food additives, such as vitamins, bioactive compounds and antimicrobials. These packages can be considered simple, composite or even made up of different layers.

Gelatine, obtained from the hydrolysis of collagen, is widely produced on the domestic market and has been studied both in the production of films using the casting technique and in the preparation of film solutions for application in different foodstuffs, given its compatibility, edibility, non-toxicity, ease of obtainment and low cost [2]. Sweet cherries were coated with an edible coating based on carboxymethyl chitosan and gelatine [3], and guavas were coated with a polymer mixture made up of natural cassava starch, casein and gelatine [4]. Mannucci et al. [5] demonstrated through the aromatic profile of Fuji apples that coating them with gelatine slowed down ripening.

The incorporation of essential oils and plant extracts with antimicrobial activity has been widely studied as an alternative to further improve the performance of edible coatings aimed at the safety and quality of meat products [6], vegetables [7] and fruits [8].

A coating of chitosan gelatine extracted from fish skin containing an edible black tea extract inhibited the decrease in weight loss and texture of papaya and suppressed microbial growth during storage [8]. The antimicrobial activity of plant extracts is assessed by determining a small amount of the substance required to inhibit the growth of the microorganism; this value is known as the Minimum Inhibitory Concentration (MIC) [9]. Concentrations of 25 and 50% of ethanolic extract of red propolis have demonstrated antimicrobial activity against *Streptococcus mutans* and *Candida albicans* [10].

Brazilian propolis is considered a food-grade ingredient for food applications due to its biological activity, which consequently generates great potential for use as an antimicrobial agent in edible gelatine-based coatings. Among the biological activities of propolis that have been highlighted in recent years are antimicrobial, antioxidant [10,11,12], antiparasitic [13,14], cytotoxic [12,13] and anticancer actions [15]. Its biological activities are attributed to the flavonoids and phenolic acids found in its composition [16].

Red propolis, whose botanical origin is *Dalbergia ecastophyllum,* is found in the northeast of Brazil. It is a resinous substance produced by Africanized bees (*Apis melifera* L.) from exudates collected from different parts of plants [17]. It is considered a lipophilic substance and is hard and brittle at low temperatures, but soft, malleable and viscous when slightly heated [18]. Propolis is composed of around 50 to 60% flavonoids and phenolic acids, 30 to 40% wax, 5 to 10% essential oils, 5% pollen, and small amounts of metals and vitamins [19].

Due to its antimicrobial effect and its profile of bioactive compounds, red propolis can be used as a bioactive ingredient in gelatine coatings to extend the shelf life of perishable foods such as grapes. Grapes are one of the fruits most consumed by the world’s population due to their attractive purple color, aroma and characteristic flavor. In addition to their sensory aspects and quality attributes, their high content of bioactive compounds, such as anthocyanins, ascorbic acid and phenolic compounds, which show high antioxidant activity, have encouraged consumers to add them to their regular diet [20,21].

However, grapes are highly susceptible to decomposition during post-harvest storage [22] due to their high nutrient content, excessive humidity, fine texture and high respiration rates. During the ripening process, changes in color, reduced texture and firmness, reduced weight, decreased nutritional value and increased microbial multiplication lead to deterioration in quality and senescence of grapes [23]. In developing countries, it is estimated that 30 to 40% of production is lost in the post-harvest, processing and distribution stages, representing a waste of resources used in production, such as land, water, energy and inputs [24].

Thus, the application of technologies is necessary to avoid reducing post-harvest losses of grapes in order to ensure that consumers can enjoy fresh fruit of good quality. In this context, the aim of this work was, firstly, to develop edible films based on gelatine and red propolis extract and to evaluate their antimicrobial effect. Subsequently, gelatine coatings incorporating propolis extract were evaluated for their ability to extend the post-harvest shelf life of grapes.

## 2. Materials and Methods

### 2.1. Materials

The materials used in the preparation of biodegradable and edible films and coatings were type A gelatine, Bloom 240, GAP 6 (Gelita do Brasil, Cotia, São Paulo, Brazil), as a polymeric material, and red propolis (Canavieiras, Bahia, Brazil), as an antioxidant and antimicrobial agent. Biodegradable and edible coatings were applied to fresh grapes (*Crimson*) purchased at the local market in Dourados, MS, Brazil. After acquiring the raw material, it was immediately taken to the Food Technology Laboratory of the Faculty of Engineering at the Federal University of Grande Dourados (UFGD) for processing. For antimicrobial analysis, *Staphylococcus aureus* (ATCC 25923), *Escherichia coli* (ATCC WDCM00013), *Pseudomonas aeruginosa* (ATCC 27853) and *Salmonella typhimurium* (ATCC WDCM 00031) were donated by SENAI (Dourados, MS, Brazil); Brain Heart Infusion Broth (BHI) (Prodimol Biotecnologia, Belo Horizonte, MG, Brazil) and Mueller–Hinton Agar (Kasvi, São José dos Pinhais, PR, Brazil) were also used.

### 2.2. Characterization of Propolis

#### 2.2.1. Determination of Bioactive Compounds

Analysis of the chemical constituents of propolis extract was performed using a GC-MS QP 2020 (Shimadzu Corp, Columbia, MD, USA). To identify the compounds, the following conditions were adopted: injector and detector temperature of 280 °C, using ultra-pure helium as carrier gas, with a column flow of 1.82 mL/min and a split of 5:1. The oven had an initial temperature of 50 °C for 3 min and was heated at a rate of 3 °C/min, with a final temperature of 280 °C for 2 min. The injection volume used was 1 µL, at a dilution of 1/100.

To prepare the samples, 1 mg of each sample was dissolved in 1.5 mL of a 1:1 methanol/dichloromethane solvent mixture. Then, 1 µL of this solution was injected into the Shimadzu Model QP2020 Gas Chromatograph coupled to a Mass Spectrometer. The column used was a 30 m DB-5 ms with a thickness of 0.25 mm and a film of 0.25 µM.

#### 2.2.2. Antioxidant Capacity

The antioxidant capacity using the DPPH-free radical method was determined according to Brand-Williams, Cuvelier and Berset [25]. Initially, a solution of the DPPH (2,2-Diphenyl-1-picrylhydrazyl) radical with an absorbance of 0.7 (0.6 mM) was prepared and determined at 517 nm in a spectrophotometer. Samples of the extract were diluted in methanol (0.3 g/mL), and aliquots (100 μL) of each dilution were added to 3.9 mL of DPPH. The solution was kept in the absence of light for a period of 30 min. The antioxidant capacity was expressed as the amount of antioxidant compounds needed to reduce the initial DPPH concentration by 50% (EC_50_).

### 2.3. Production of Films for Antimicrobial Activity (“Halo Test”)

The base film solution was obtained by hydrating the gelatine (10%) for 1 h in cold water, after which the solution was heated in a lab water bath (Nova Orgânica, São Paulo, Brazil) at 70 °C for ten minutes [26]. After the gelatine had completely dissolved, the films were prepared using the casting technique, with the addition of 5, 10, 15, 20 and 25% red propolis extract, under stirring and controlled heating at 30 °C. The film solutions were then dispersed on acrylic plates (12 cmdiameter) and dried for 24 h at 30 °C in an air-circulating oven (Marconi, MA 037, Piracicaba, SP, Brazil). After drying, the films were removed from the acrylic sheets and analyzed visually (color homogeneity, presence of bubbles and particles) and tactilely (handling).

The antimicrobial activity of the films was determined by the “Halo Test” using 5.5 mm diameter discs which were placed on Mueller–Hinton agar plates containing bacteria inoculated in the exponential growth phase: *Staphylococcus aureus*, *Escherichia coli*, *Pseudomonas aeruginosa* and *Salmonella typhimurium*, according to the methodology standardized by the National Committee for Clinical Laboratory Standards [27]. The inoculated plates were incubated in a Bacteriological Oven (Solab, SL-101, Piracicaba, SP, Brazil) at 35 ± 2 °C for 24 h. After this period, microbial growth was checked, and the degree of inhibition was expressed as a halo of inhibition (mm) around the disks.

### 2.4. Production of Film Solutions to Cover the Grapes

The film solutions were prepared in accordance with Section 2.3. The red propolis extract was added directly, and the concentration of the extract was defined according to the result obtained in the “halo test”, using only the formulations that showed antimicrobial activity (formation of an inhibition halo ≥8 mm). To cover the grapes, 4 gelatine-based formulations were made: (i) pure gelatine, without plasticizer, at room temperature; (ii) gelatine with 25% red propolis extract, at room temperature; (iii) pure gelatine, without plasticizer, at refrigeration temperature; (iv) gelatine with 25% red propolis extract, at refrigeration temperature. For the addition of 25% propolis extract, the film solution was cooled to 30 °C.

### 2.5. Preparation of Grapes and Application of Film Solutions

The grapes were washed under running water and kept in an 8% sodium hypochlorite solution for 10 min. After sanitization, the fruit was dried at room temperature and immersed in the film solutions prepared in Section 2.4. The fruit was fully immersed in the solution for 1 min and dried for approximately 10 min at room temperature. The temperature of the film solution was kept at 30 °C. Fruit samples without coatings were also prepared and stored for 25 days under refrigeration in a BOD oven (Tecnal, Niort, France, TE-391) at 5 °C ± 2 °C and for 19 days at room temperature.

### 2.6. Characterization of the Grapes

#### 2.6.1. Visual Appearance and Color Parameters

The grape samples were analyzed for appearance by assessing the gloss and visual adherence of the coating, as well as their quality by the absence of physiological defects, fungal deterioration, holes and rot. The color of the grape samples was determined using a colorimeter (Konica Minolta, Tokyo, Japan/CR-400/410) through the parameters of luminosity (L*), chroma a* (parameter of color variation from green to red) and chroma b* (parameter of color variation from blue to yellow). The readings of all the parameters were taken in triplicate.

#### 2.6.2. Loss of Mass

To determine mass loss, the fresh and covered grape samples were initially weighed on day 0 on an analytical balance (Ohaus, PA214CP, Barueri, SP, Brazil). Subsequently, on days 1, 4, 10, 15, 19 and 25, the mass loss was monitored by subtracting the initial and final weight of the fruits, with the results expressed in %. All the analyses were carried out in triplicate.

#### 2.6.3. Determining pH

The pH was determined using a pH meter (PH–2000, Instrutherm, Tatuapé, SP, Brazil), duly calibrated. Samples of 3 g of grapes homogenized in a domestic blender were previously diluted in distilled water [28]. The contents were stirred and homogenized using a glass rod until the particles were uniform enough to measure the pH. All pH analyses were carried out in triplicate.

#### 2.6.4. Total Titratable Acidity (TTA)

The total titratable acidity (TTA) was determined by titrating a 10 g sample of grape crushed pulp and homogenizing it (in a domestic blender) with 90 mL of distilled water using a 0.1 mol·L^−1^ NaOH solution, using 1% phenolphthalein as an indicator, with the results expressed as % citric acid [29]. All the analyses were carried out in triplicate.

#### 2.6.5. Total Soluble Solids (TSS)

The total soluble solids (TSS) contents of the homogenized (in a domestic blender) grape samples were measured directly in a digital refractometer (DIGITAL ATAGO, PAL-SALT, Ribeirão Preto, SP, Brazil), with the results expressed in °Brix. All analyses were carried out in triplicate.

#### 2.6.6. Total Solids

The total solids of the grape samples were determined using the gravimetric method in an air-circulating oven (Lucadema, LUCA-82/250, São José do Rio Preto, SP, Brazil) at 105 °C [29]. All the analyses were carried out in triplicate.

#### 2.6.7. Sensory Evaluation

The grape samples, with and without toppings, stored under refrigeration (5 °C), were evaluated after 25 days using a 9-point structured hedonic scale, ranging from “extremely liked” with a score of 9 to “extremely disliked” with a score of 1, according to the methodology described by Dutcosky [30]. A total of 55 untrained tasters aged between 18 and 39 took part in the sensory analysis. They evaluated the attributes of brightness, color, overall appearance and intention to buy. The samples were coded with 3 digits, placed on disposable white plates and presented to the tasters monadically (one by one); block balancing was carried out to avoid the first-order carry-over effect, according to Macfie et al. [31]. The acceptability index calculated for the three formulations is described in Equation (1).

(1) $$\mathrm{Acceptability}\mathrm{index}\mathrm{percentage}\left(IA\%\right)=\frac{\mathrm{average}\mathrm{score}\mathrm{obtained}\mathrm{for}\mathrm{the}\mathrm{product}}{\mathrm{maximum}\mathrm{score}\mathrm{given}\mathrm{to}\mathrm{the}\mathrm{product}}\times 100$$

### 2.7. Statistical Analysis

InfoStat^®^ software (version 2018d) was used to calculate the analysis of variance (ANOVA). Tukey’s test was performed to determine the difference between the means at a 95% confidence level.

## 3. Results and Discussion

### 3.1. Characterization of the Bioactive Compounds and Antioxidant Activity of Red Propolis Extract

Table 1 shows the results for the composition of the red propolis extract. Twenty compounds were identified, among which lupeol, octomethyl, pentamethyl and copaene were considered the main compounds found in red propolis extract, as they are the compounds with the highest percentages of peak area. This is because the area under a peak (peak area count) is an estimate of the concentration of the compound it represents. In the evaluation of Brazilian red propolis samples carried out by Aldana-Mejía et al. [32], the main compounds identified were vestitol, medicarpin and neovestitol. Similarly, medicarpin, an isoflavonoid that belongs to a group of flavonoids, was also found in the propolis extract in this study.

According to Watanabe et al. [33], phenolic acids and their esters, flavonoids (flavones, flavonones, flavonols, dihydroflavonols and chalcones), terpenes, aromatic aldehydes, alcohols, fatty acids, stilbenes and β-steroids are the main groups of bioactive compounds found in propolis.

Red propolis extract has a high content of phenolic compounds and flavonoids [34]. Gomes Sá et al. [35] found a total content of phenolic compounds and flavonoids ranging from 21.72 to 1.79 mg GAE/dry matter and 8.21 to 0.31 mg EQ/dry matter, respectively, in particles loaded with red propolis extract. The variation in the composition of bioactive compounds identified in propolis depends on the resins and balsams of the plants from which bees’ propolis comes [36].

According to Aldana-Mejía et al. [32], red propolis has a diverse chemical composition, with red propolis mainly composed of isoflavonoids (medicarpin, formononetin and vestitol, among others). Guzelmeric et al. [36] evaluated 47 propolis samples from different regions, and the main compounds identified were caffeic acid, quercetin, 3-MQ, apigenin, kaempferol, chrysin, pinocembrin, galangin and CAPE in the propolis samples, the concentrations varying according to the region where the propolis was obtained. However, regardless of the region where red propolis is obtained, it has several active compounds of commercial interest, with proven benefits for human health.

Previous studies have shown that the phenolic compounds found in propolis extract have high biological activity, including the capture of reactive and specific species [37]. Thus, as expected, the red propolis extract showed a high capacity for eliminating DPPH radicals. The concentration of the extract needed to inhibit 50% of DPPH radicals (CE_50_) was low at 0.0233 ± 0.0006 g mL^−1^, which shows its high antioxidant activity. Even so, the value found in this study is still higher than that reported by Lima et al. [38], who found a range of 0.96–3.59 (DPPH CE_50_ mg propolis mL^−1^).

According to Cai et al. [39], the configuration and number of hydroxyl groups in the aromatic rings, as well as the configuration of other substituents, such as glycosylation, in the structure of phenolic compounds directly influence their activity in eliminating reactive species.

### 3.2. Antimicrobial Activity of the Films (“Halo Test”)

**Figure 1:**
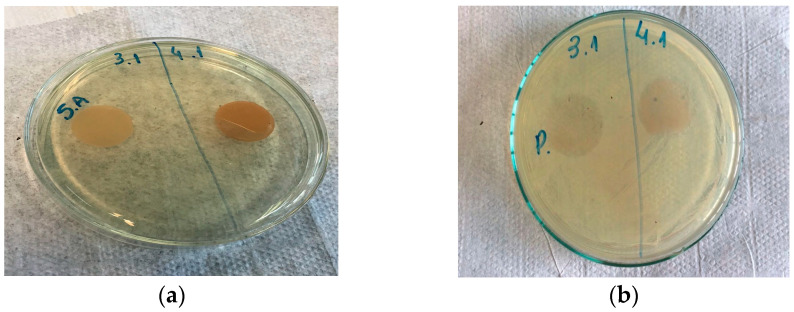
Antimicrobial activity of films with red propolis extract against (**a**) *Staphylococcus aureus* and (**b**) *Pseudomonas aeruginosa*.

The antimicrobial activity of the films containing red propolis extract was assessed using the disk diffusion test on solid media against *Staphylococcus aureus*, *Escherichia coli*, *Pseudomonas aeruginosa* and *Salmonella typhimurium* (Figure 1).

The films with 15 and 25% red propolis extract showed antimicrobial activity against the bacteria *Staphylococcus aureus* and *Pseudomonas aeruginosa*, respectively. In Figure 1a, in the film with 15% propolis, an inhibition halo of 24 mm was formed against the Gram-positive bacterium *S. aureus* and against the Gram-negative bacterium *P. aeruginosa,* and the halo formed in the film with 25% propolis was 27 mm (Figure 1b). However, no inhibition halos were observed against the *Escherichia coli* and *Salmonella typhimurium* bacteria studied. According to Ponce et al. [40] and Palmeira et al. [41], to be considered a satisfactory inhibitory activity, the inhibition halo must have a diameter ≥8 mm. Thus, the concentrations of 15 and 25% propolis extract were minimally inhibitory against *S. aureus* and *P. aeruginosa,* respectively. However, only the 25% concentration of propolis extract was considered for incorporation into the films to cover the grapes, as it was able to inhibit both bacteria tested, as it had a greater halo of inhibition.

The mechanism of action of propolis is not yet fully understood, but some studies suggest that the constituents of propolis interfere with cell division, causing disorganization of the cytoplasm, inhibiting protein synthesis and causing cell death [42]. Propolis shows efficient activity against Gram-positive bacteria and reduced activity against Gram-negative bacteria [43,44].

The cell wall of Gram-positive bacteria is made up of teichoic and lipoteichoic acids, which can help penetrate the hydrophobic components of essential oils into the cells [45], while Gram-negative bacteria have a lipopolysaccharide (LPS) outer membrane, which replaces most of the phospholipids in the outer membrane and limits the diffusion rate of hydrophobic compounds in the polysaccharide layer [46]. For this reason, Gram-negative bacteria are more resistant to the effects of essential oils than Gram-positive ones [47]. In spite of this, in the present study, we obtained inhibition halos for both *S. aureus* (Gram-positive bacteria) and *P. aeruginosa* (Gram-negative bacteria).

Propolis and its extracts also have a broad spectrum of antimicrobial activity against a variety of bacteria, fungi, parasites and viruses [48,49,50,51]. With the aim of formulating safe products in the cosmetics industry and preserving the final products, Packer et al. [52] tested the bacteriostatic and fungistatic activities of copaiba, rosemary, Melaleuca, garlic, andiroba and propolis oils against strains of *Staphylococcus aureus* (ATCC 6538), *Escherichia coli* (ATCC 8739), *Pseudomonas aeruginosa* (ATCC 9027) and *Candida albicans* (ATCC 10231). Among the samples analyzed, andiroba, copaiba and garlic oils showed no bacteriostatic or fungistatic activity against the microorganisms tested, and propolis showed an inhibition halo for *S. aureus* (Gram-positive bacteria) and *E. coli* (Gram-negative bacteria) and no inhibition halo for *P. aeruginosa* (Gram-negative bacteria). The effective inhibition of *P. aeruginosa* by the red propolis extract tested is a promising result, given that this microorganism is a member of the most important group of spoilage bacteria in refrigerated fresh foods [53].

### 3.3. Characterization of the Film Added with Red Propolis Extract

#### Visual Assessment

**Figure 2:**
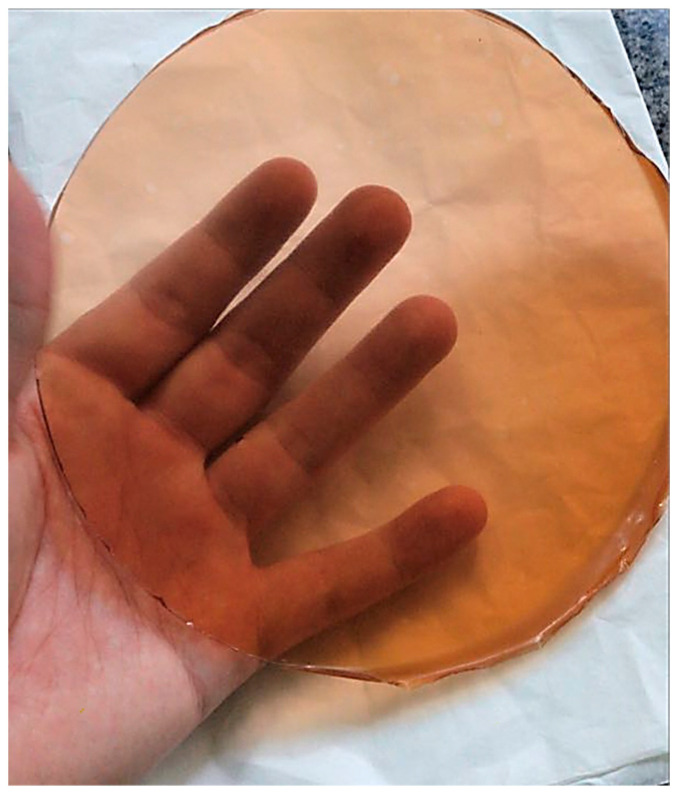
Gelatine film incorporated with 25% red propolis extract.

Figure 2 shows one of the films made from gelatine with 25% red propolis extract incorporated into it. The films produced had a reddish color; a homogeneous, transparent surface; no insoluble particles; and no apparent cracks, suggesting that the propolis extract, at the concentration studied, has plasticizing capacity for this macromolecule, and that it is not necessary to use a plasticizer in the film formulation. Alves et al. [54] reported that cassava starch films without glycerol were fragile and had low mechanical properties. Gheribi et al. [55] reported the need to add plasticizer to cactus mucilage-based films in order to obtain films with good handling properties and appearance. According to Bertuzzi et al. [56], the addition of a plasticizing agent to edible films is necessary to reduce their fragility.

After the drying process, all the films had a shiny side (facing the plate) and a dull side (facing the air). Gloss is a surface property and is related to the morphology of the film surface, which varies according to exposure and contact with the air during the drying process [57]. According to Silva et al. [58] and Villalobos et al. [57], the gloss depends on the degree of polishing, i.e., the greater the surface roughness, the lower the gloss of the films. Considering that this film formulation will be used as a topping, the shine characteristic observed can be an attraction for consumers, as it will impact on the appearance of the grapes it covers.

### 3.4. Characterization of the Grapes Covered with the Film Solution

#### 3.4.1. Visual Appearance and Color Parameters

**Figure 3:**
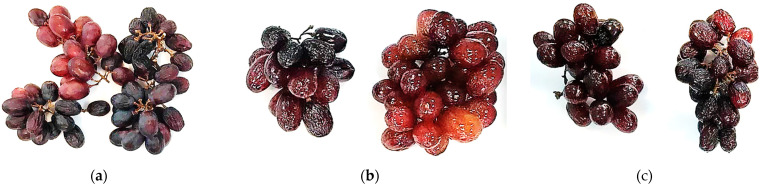
Grapes after 19 days of storage at room temperature (25 ± 2 °C): control sample (**a**), grapes covered with gelatine (**b**) and grapes covered with gelatine and propolis (**c**).

**Figure 4:**
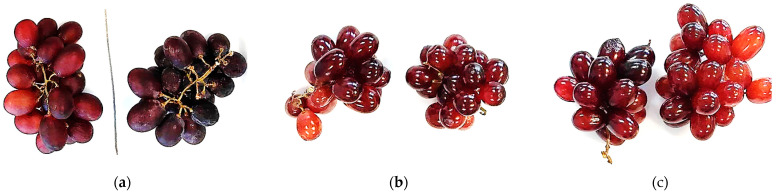
Grapes after 25 days of storage at refrigeration temperature (5 ± 2 °C): control sample (**a**), grapes covered with gelatine (**b**) and grapes covered with gelatine and propolis (**c**).

The visual appearance of the gelatine-coated, gelatine-coated with red propolis extract, and uncoated grapes during shelf life and storage at 25 °C and 5 °C can be seen in Figure 3 and Figure 4, respectively. It is clearly visible that the coated grapes looked better after 25 days of refrigerated storage compared to the grapes stored at room temperature after 19 days.

It can be seen that the uncoated grapes appeared dull in color and shriveled and wrinkled, especially when stored at room temperature. In contrast, the grapes coated with gelatine (Figure 3 and Figure 4b) and with the gelatine coating containing red propolis extract (Figure 3 and Figure 4c) had a brighter appearance and visual turgor. Similarly, Chen et al. [59] reported that grapes with a glucomannan/curdlan konjac-based coating with and without camellia oil incorporated into it showed no shriveling. However, the texture of the control group was soft, and there were shrinkage of the skin and disease spots after 6 days.

Therefore, the gelatine coating and gelatine containing red propolis extract were able to inhibit the loss of water from the grapes, maintain their color and delay their decomposition, extending their shelf life.

In addition, it can be seen from the color parameters in Table 2 that the edible coatings applied to the surface of the grapes did not cause any undesirable visible changes to their color, and they were therefore considered effective in maintaining color. The results of the statistical tests showed that the coatings had no statistically significant effect (*p* > 0.05) on the level of variation in the a* and b* values during the storage period at 25 and 5 °C. However, the luminosity of the grapes decreased in all treatments during storage, and there was a significant decrease between the observations on day 0 and day 25 (*p* < 0.05) in the L* values of all treatments, regardless of the presence or absence of the coating when the grapes were stored at 5 °C.

The grapes with the coating had greater luminosity than the grapes without the coating. The results of the present research are consistent with previous findings by Zhang et al. [3], who reported that sweet cherries coated with an edible coating obtained from a mixture of carboxymethyl chitosan and gelatine with calcium chloride and ascorbic acid obtained higher L*, chroma and hue angle values, exhibiting better skin color throughout the storage period. Fruit color is one of the most important characteristics related to quality and freshness, and for this reason it has a great influence on the acceptability of fruit to consumers [8].

#### 3.4.2. Loss of Mass

Fruit loses mass during storage due to natural processes, such as respiration, transpiration and the loss of volatile and aromatic compounds [7]. The physiological process of transpiration involves the evaporation of water through a fruit’s anatomical structures into the environment [60]. This is affected by the characteristics of the environment, such as ambient temperature and relative humidity and air circulation, as well as by the characteristics of the fruit, such as its respiration rate, size, shape and surface area, skin permeability, and maturity. This external water transfer from the fruit causes changes in its weight and appearance, resulting in a shriveled, wrinkled appearance; altered texture, color and flavor; and/or loss of nutritional value, which consequently leads to a decline in its quality and rejection by the consumer. This is why techniques to reduce this loss of mass are so sought after.

**Figure 5:**
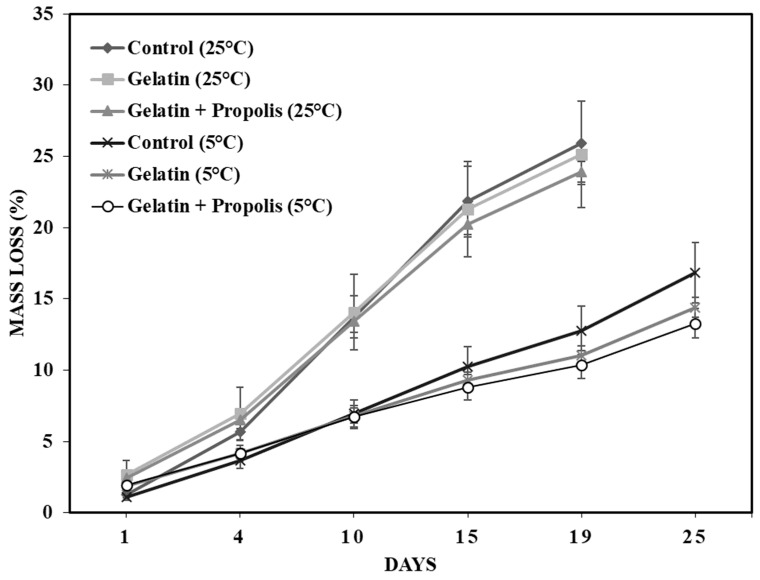
Mass loss of fresh grapes in the control group (without coating), the gelatine group and the gelatine + propolis group conditioned at room temperature (25 ± 2 °C) and refrigerated (5 ± 2 °C).

Although it was observed that the percentage mass loss of all the samples increased gradually, it can be seen in Figure 5 that the lowest mass loss values were found for the grapes stored under refrigeration when compared to those stored at room temperature. This is because low temperature causes a reduction in metabolic activity and chemical changes slow down, which means that the respiratory process and transpiration are reduced [8,61].

In addition, the grapes coated with gelatine and with gelatine containing propolis extract had significantly lower mass loss (*p* < 0.05) when compared to the uncoated grapes (Figure 5). Furthermore, the group of grapes coated with gelatine containing red propolis extract had the lowest weight loss when stored at both 25 °C and 5 °C. The total weight of the control group was reduced by 25.95 ± 2.93% after 19 days of storage at 25 °C, and the reduction in mass was 25.16 ± 3.73% and 23.92 ± 0.74% for the grapes coated with gelatine and gelatine with propolis extract, respectively. For the control grape samples kept at a temperature of 5 °C, the loss of mass reached 16.84 ± 2.10% after 25 days, while for the fruit coated with gelatine and gelatine with propolis extract, these values were only 14.39 ± 0.7% and 13.27 ± 1.03%, respectively. These behaviors are in line with those found by Fakhouri et al. [62], who reported that the addition of gelatine to films used to cover crimson red grapes resulted in a lower loss of mass when compared to samples without coatings.

According to Souza et al. [63], an edible coating made up of a combination of alginate (2.0%), galactomannan (0.5%), cashew gum (0.5%) and gelatine (2.0%) reduced the mass loss of ‘Italia’ grapes and maintained their firmness and color, even after 9 days of storage, compared to the control. According to the authors, this combination of materials with the gelatinous matrix formed a better protective barrier against temperature and was capable of reducing the weight loss of the grapes.

Similarly, the metabolic activity and action of the enzymes responsible for the degradation of the mass and firmness of tomatoes was limited due to the alteration of the permeability of the fruit by a coating of gelatine and açaí oil [7]. Chen et al. [59] reported that the addition of camellia oil to a glucomannan/curdlan konjac-based coating improved the water barrier properties of this coating by completely blocking the escape of water from the grapes but at the same time providing intermolecular channels in the polymer matrix to ensure a low level of water vapor transmittance. In view of this, it is believed that, in this work, the combination of gelatine with red propolis extract, a fat-soluble extract, generated a less permeable barrier on the surface of the grapes, which inhibited the transpiration process when compared to the coating composed only of gelatine, which is a water-soluble material. However, in a previous study by Pastor et al. [64], no clear differences in weight loss were detected between grape samples coated with hydroxypropylmethylcellulose with or without propolis. This may be due to the differences between the materials used to make the coatings and the extensibility of the coatings on the skin of the fruit and the consequent degree of uniformity of the coatings.

#### 3.4.3. Determination of pH, Total Titratable Acidity, Total Soluble Solids and Total Solids

Table 3 shows the results of the physicochemical analysis of the grapes with and without the topping. Statistically significant differences (*p* < 0.05) were observed for pH, total titratable acidity, total soluble solids and total solids between treatments and storage times and temperatures.

The pH and total titratable acidity of the control sample on day 0 were 3.38 and 0.66 (citric acid g 100 mL^−1^), and after 19 days stored at 25 °C and 5 °C the pH levels were 3.26 and 3.28 and the acidities were 0.94 and 0.84 (citric acid g 100 mL^−1^), respectively. Meanwhile, in the samples of grapes coated with gelatine and with gelatine with propolis extract, the pH levels on day 19 at 25 °C were 3.55 and 3.44, respectively, and at 5 °C, they were 3.36. The acidities for these samples were 0.86 and 0.75 citric acid g 100 mL^−1^ when stored at 25 °C and 0.72 and 0.81 citric acid g 100 mL^−1^ when stored at 5 °C. Souza et al. [63] reported that the pH values for all groups of grapes with and without edible coatings ranged from 2.93 to 3.58 during 12 days of storage, which values are close to those found in the present study.

The coating and storage at room temperature provided a higher pH value than the uncoated grapes. As for acidity, soluble solids content and total solids, although a gradual increase was observed in the coated fruit at the end of the experiment compared to day 0, this was not as high when compared to the control group, especially when stored at 5 °C. This proves that the gelatine coating and gelatine containing propolis extract were effective in delaying the ripening of the grapes.

Coverings and low temperatures tend to decrease the respiration rate, which means that the anabolic and catabolic activities that take place during fruit ripening are reduced. During the fruit ripening process, starch is broken down into glucose by glycolysis. This is followed by the production of CO_2_, acids, heat and water in the Krebs cycle by the aerobic transformation of pyruvic acid and other organic acids, which has an impact on pH values, acidity, soluble solids and water content [65].

Total soluble solids and total solids increased during storage of the grapes from around 19 °Bx and 19.72% on day 0 to around 21 °Bx and 20.88% on the 15th day of storage at 25 °C, respectively. The observed increase in soluble solids content occurred as the fruit ripened due to the increased bioconversion of sucrose into glucose, which results in the accumulation of free sugars [7,66]. Sugars and organic acids are the main constituents of the soluble solids present in fruit, which are responsible for the flavor and consequent acceptance of fresh fruit by consumers, as well as influencing the chemical characteristics of the food, such as pH, total acidity, sweetness and microbial stability [67]. Citric, malic and tartaric acids are the main ones found in grapes [68]. In addition, total acidity tends to increase in the initial period of ripening and decreases in the final period of ripening towards senescence.

It has been reported that coating grapes with polymalate caused no obvious changes in total soluble solids, titratable acid content and soluble sugar content during storage [69]. Fluctuations in soluble solids and water content are due to metabolic reactions that occur during fruit ripening. Therefore, the assessment of pH, acidity and soluble solids during the storage period is extremely important for evaluating the quality of fruit and its stage of ripeness for processing.

#### 3.4.4. Sensory Evaluation

It was not possible to carry out a sensory evaluation of the grapes stored at room temperature after 25 days because they had completely deteriorated. The sensory evaluation of the grapes stored at 5 °C after 25 days is shown in Table 4. It can be seen that the average scores obtained in the evaluation of the uncoated grapes for the overall appearance and brightness attributes were between 2 and 3, which means “disliked very much” and “disliked moderately”, while, for the color attribute, the average score was between 3 “disliked moderately” and 4 “disliked slightly”. These results show that the evaluators disliked the appearance, color and brightness of the uncoated grapes, which resulted in acceptance rates below 43%.

On the other hand, there was a significant increase in the average scores for the grapes coated with gelatine and gelatine with propolis extract for overall appearance, color and brightness attributes, ranging from 7 “I liked it slightly” to 8 “I liked it a lot”. In addition, the acceptability index of the grapes with coatings rose to over 78% for all the attributes evaluated. Fakhouri et al. [62] also obtained higher scores for grapes coated with starch and gelatine mixtures for overall appearance and for purchase intention compared to the control group.

The additional shine provided by coating the fruit was also well accepted by the evaluators. According to Teixeira, Meinert and Barbetta [70], for a product to be defined as sensorially accepted in relation to its attributes, it must achieve an acceptability index of at least 70% or an average score greater than or equal to 7. Given this, it is clear that the coated grapes were accepted and approved sensorially by the tasters.

**Figure 6:**
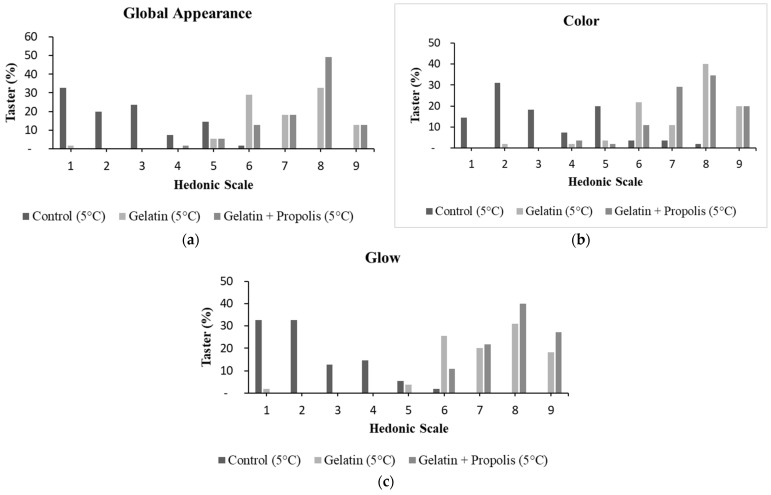
Sensory evaluation of grapes, using a hedonic scale for global appearance (**a**), color (**b**) and glow (**c**). Avaliation of of 55 consumers for each sample. Scores for overall appearance, color and glow: 1 = disliked extremely; 2 = disliked very much; 3 = disliked moderately; 4 = disliked slightly; 5 = liked/disliked; 6 = liked slightly; 7 = liked moderately; 8 = liked very much; 9 = liked extremely.

Furthermore, the addition of red propolis extract to the gelatine coating had a positive impact on the acceptability of the grapes when compared to the gelatine coating. For the overall appearance and shine attribute, 62% and 67% of the evaluators said they liked the grapes coated with gelatine and propolis extract very much to extremely, while only 45% and 49% of them said this for the grapes coated with gelatine, respectively (Figure 6a,c). On the other hand, when evaluating the uncoated grapes, it can be seen that none of the evaluators said they liked their appearance or shine very much or at all, and only 2% of the evaluators said they liked their color very much.

Among the most important quality factors for food is its overall appearance and color, since they reflect sensory attractiveness or rejection, especially for fresh fruit. It can therefore be concluded that edible coatings can make a positive contribution to the sensory acceptance of fresh grapes during their shelf life.

## 4. Conclusions

Red propolis extract is a rich source of bioactive compounds with high antioxidant activity. Incorporating propolis extract at a concentration of 25% into gelatine films showed an antibacterial effect against *Staphylococcus aureus* and *Pseudomonas aeruginosa*. Coating grapes with gelatine and a gelatine coating containing propolis extract allowed them to maintain their color and generated a shiny appearance on their surface, reduced mass loss during storage, and prevented the fruit peels from shriveling and wrinkling compared to the control group. In addition, the gelatine coating containing propolis extract associated with refrigeration temperature had the greatest potential to slow down the ripening process of the grapes while maintaining their physical and chemical properties. In the sensory analysis, the coated grapes were more acceptable in terms of overall appearance, color and brightness than the uncoated grapes. Therefore, the gelatine coating and the gelatine containing red propolis extract had a positive effect on the shelf life of fresh grapes, preserving their quality and improving their sensory acceptability during 25 days of storage.

## Figures and Tables

**Table 1:** Identification of the individual compounds present in the red propolis extract.

Peak	R. Time	Area	Area (%)	Name
1	25.854	5369065	7.23	Copaene
2	27.529	1129858	1.52	Bicyclo[3.1.1]hept-2-ene, 2,6-dimethyl-6-(4-methyl-3-pentenyl)-
3	31.356	867710	1.17	.beta.-Bisabolene
4	31.900	1126380	1.52	Naphthalene, 1,2,3,5,6,8a-hexahydro-4,7-dimethyl-1-(1-methylethyl)-, (1S-cis)-
5	42.392	1261623	1.70	Squalene
6	48.530	1007511	1.36	Hexadecanoic acid, ethyl ester
7	64.373	1637516	2.20	Medicarpin
8	67.982	2073647	1.67	Phenol, 2-(3,4-dihydro-7-methoxy-2H-1-benzopyran-3-yl)-5-methoxy-
9	69.216	1456579	2.79	2H-1-Benzopyran-7-ol, 3,4-dihydro-3-(2-hydroxy-4-methoxyphenyl)-
10	70.029	6964543	1.96	Octadecanoic acid, ethyl ester
11	72.875	6964543	9.38	2,6,10,14,18-Pentamethyl-2,6,10,14,18-eicosapentaene
12	73.095	4616562	6.21	2,6,10,14,18-Pentamethyl-2,6,10,14,18-eicosapentaene
13	74.671	4507181	6.07	2,6,10,14,18-Pentamethyl-2,6,10,14,18-eicoosapentaene
14	81.393	2096977	2.82	4,4,6a,6b,8a,11,11,14b-Octamethyl-1,4,4a,5,6,6a,6b,7,8,8a,9,10,11,12,12a,14,14a,14b-octadecahydro-2H-picen-3-one
15	82.102	12326129	16.59	4,4,6a,6b,8a,11,11,14b-Octamethyl-1,4,4a,5,6,6a,6b,7,8,8a,9,10,11,12,12a,14,14a,14b-octadecahydro-2H-picen-3-one
16	83.411	13730291	18.48	Lupeol
17	83.667	4208244	5.66	D:B-Friedo-B’:A’-neogammacer-5-en-3-ol,
18	85.278	2164620	2.91	4,4,6a,6b,8a,11,11,14b-Octamethyl-1,4,4a,5,6,6a,6b,7,8,8a,9,10,11,12,12a,14,14a,14b-octadecahydro-2H-picen-3-one
19	86.695	3058562	4.12	Urs-12-en-3-ol, acetate, (3.beta.)-
20	86.871	3440076	4.63	Lup-20(29)-en-3-ol, acetate, (3.beta.)-

**Table 2:** Color parameters of grapes with different concentrations of edible coating stored for up to 19 days at 25 °C and 25 days at 5 °C.

Parameters	Control (25 °C)	Gelatine (25 °C)	Gelatine + Propolis (25 °C)	Control (5 °C)	Gelatine = (5 °C)	Gelatine + Propolis (5 °C)
**Day 0**						
L*	28.89 ± 2.5 Ba*	28.89 ± 2.5 Aa	28.89 ± 2.5 Aa	28.89 ± 2.5 Ba	28.89 ± 2.5 Ba	28.89 ± 2.5 Ba
a*	6.36 ± 1.8 Aa	6.36 ± 1.8 Aa	6.36 ± 1.8 Aa	6.36 ± 1.8 Aa	6.36 ± 1.8 ABa	6.36 ± 1.8 Aa
b*	2.52 ± 1.7 Aa	2.52 ± 1.7 Aa	2.52 ± 1.7 Aa	2.52 ± 1.7 Aa	2.52 ± 1.7 Aa	2.52 ± 1.7 Aa
**Day 01**						
L*	27.27 ± 1.3 ABbc	27.92 ± 3.5 Ac	27.27 ± 3.1 Abc	22.59 ± 1.4 Aab	21.95 ± 2.0 Aa	23.38 ± 5.6 Aabc
a*	4.83 ± 2.0 Aa	5.93 ± 1.6 Aab	5.40 ± 1.2 Aa	8.10 ± 1.9 Ab	6.81 ± 1.1 Bab	5.28 ± 1.3 Aa
b*	3.68 ± 2.2 Aa	2.00 ± 08 Aa	2.21 ± 1.1 Aa	3.40 ± 1.1 Aa	2.77 ± 0.7 Aa	2.95 ± 0.9 Aa
**Day 04**						
L*	25.53 ± 5.4 Aba	27.87 ± 4.6 Aa	27.35 ± 2.7 Aa	28.47 ± 1.0 Ba	28.27 ± 2.6 Ba	27.96 ± 1.8 ABa
a*	6.19 ± 2.1 Aab	5.83 ± 1.6 Aab	5.00 ± 0.76 Aab	6.46 ± 1.0 Ab	5.42 ± 0.7 ABab	4.39 ± 0.7 Aa
b*	2.08 ± 0.7 Aa	2.11 ± 1.0 Aa	2.31 ± 0.6 Aa	2.58 ± 0.7 a	1.96 ± 0.7 Aa	1.74 ± 0.5 Aa
**Day 10**						
L*	26.10 ± 2.9 ABab	27.69 ± 1.6 Abc	29.71 ± 1.3 Ac	23.69 ± 1.1 Aa	24.94 ± 2.2 Aab	25.76 ± 1.8 ABab
a*	4.87 ± 1.2 Aa	5.77 ± 1.3 Aa	5.79 ± 1.3 Aa	7.10 ± 2.0 Aa	5.47 ± 1.3 ABa	4.86 ± 1.6 Aa
b*	1.94 ± 0.8 Aa	2.10 ± 0.8 Aa	2.43 ± 0.8 Aa	3.13 ± 1.3 Aa	2.09 ± 0.6 Aa	2.38 ± 1.2 Aa
**Day 15**						
L*	22.26 ± 4.0 Aa	28.07 ± 1.4 Ab	27.85 ± 3.8 Ab	22.06 ± 1.2 Aa	24.20 ± 1.7 Aab	26.31 ± 1.3 ABb
a*	5.07 ± 2.3 Aa	5.99 ± 1.4 Aa	5.85 ± 0.9 Aa	6.86 ± 1.2 Aa	4.77 ± 0.7 Aa	4.95 ± 1.8 Aa
b*	2.40 ± 1.8 Aa	2.35 ± 0.6 Aa	2.82 ± 0.7 Aa	3.15 ± 1.0 Aa	1.90 ± 0.3 Aa	2.97 ± 2.3 Aa
**Day 19**						
L*	24.80 ± 3.9 ABab	27.46 ± 3.0 Ab	25.75 ± 2.9 Aab	21.80 ± 1.9 Aa	22.49 ± 1.7 Aa	24.27 ± 1.6 ABab
a*	5.42 ± 1.6 Aab	5.90 ± 1.6 Aab	5.45 ± 1.1 Aab	7.11 ± 1.6 Ab	5.28 ± 1.4 ABab	4.58 ± 1.6 Aa
b*	2.34 ± 1.0 Aa	2.40 ± 0.7 Aa	2.53 ± 0.7 Aa	3.40 ± 1.2 Aa	2.28 ± 0.9 Aa	2.11 ± 1.0 Aa
**Day 25**						
L*	-	-	-	23.09 ± 1.1 Aa	24.17 ± 2.1 Aa	23.03 ± 4.7 Aa
a*	-	-	-	6.97 ± 1.5 Aa	4.90 ± 1.4 ABa	5.27 ± 1.7 Aa
b*	-	-	-	2.97 ± 1.5 Aa	2.27 ± 0.5 Aa	3.50 ± 2.5 Aa

* Means and standard deviations followed by the same uppercase letter in a column and the same lowercase letter in a row did not differ statistically from each other by the Tukey test at *p* > 0.05.

**Table 3:** Determination of pH, total titratable acidity, total soluble solids and total solids of grapes with different concentrations of edible coating stored for up to 19 days at 25 °C and 25 days at 5 °C.

Formulations	pH (Decimal)	Titratable Total Acidity in Citric Acid (g 100 mL^−1^)	Total Soluble Solids (°Bx)	Total Solids (%)
**Day 0**				
Control (25 °C)	3.38 ± 0.0 Ba*	0.66 ± 0.0 Aa	19.00 ± 0.0 Aa	19.72 ± 0.1 Aa
Gelatine (25 °C)	3.38 ± 0.0 Aa	0.66 ± 0.0 ABa	19.00 ± 0.0 Ba	19.72 ± 0.1 Ba
Gelatine + Propolis (25 °C)	3.38 ± 0.0 Aa	0.66 ± 0.0 ABa	19.00 ± 0.0 Ca	19.72 ± 0.1 Ba
Control (5 °C)	3.38 ± 0.0 Ba	0.66 ± 0.0 Aa	19.00 ± 0.0 ABa	19.72 ± 0.1 ABa
Gelatine (5 °C)	3.38 ± 0.0 ABa	0.66 ± 0.0 Aa	19.00 ± 0.0 Aa	19.72 ± 0.1 Aa
Gelatine + Propolis (5 °C)	3.38 ± 0.0 Aa	0.66 ± 0.0 Aa	19.00 ± 0.0 Ba	19.72 ± 0.1 Ba
**Day 01**				
Control (25 °C)	3.63 ± 0.0 DEab	0.69 ± 0.0 Abc	18.95 ± 0.0 Ab	19.55 ± 0.2 Aab
Gelatine (25 °C)	3.71 ± 0.0 Cbc	0.75 ± 0.0 Bd	19.00 ± 0.0 Bb	19.43 ± 0.3 Bab
Gelatine + Propolis (25 °C)	3.76 ± 0.1 Ec	0.67 ± 0.0 ABCab	18.20 ± 0.0 Ba	18.79 ± 0.3 Aba
Control (5 °C)	3.55 ± 0.0 Da	0.70 ± 0.0 ABc	19.00 ± 0.6 ABb	19.96 ± 0.3 BCbc
Gelatine (5 °C)	3.60 ± 0.0 Ea	0.69 ± 0.0 Aabc	19.50 ± 0.0 Bc	21.27 ± 0.6 Ad
Gelatine + Propolis (5 °C)	3.58 ± 0.0 Ca	0.66 ± 0.0 Aa	19.50 ± 0.0 Cc	20.67 ± 0.1 Bcd
**Day 04**				
Control (25 °C)	3.50 ± 0.0 Cbc	0.66 ± 0.0 Aa	21.50 ± 0.0 Ca	19.71 ± 0.5 Ab
Gelatine (25 °C)	3.37 ± 0.0 Aa	0.74 ± 0.0 Bb	19.00 ± 0.0 Bb	17.04 ± 0.5 Aa
Gelatine + Propolis (25 °C)	3.50 ± 0.0 BCbc	0.65 ± 0.0 AB	19.95 ± 0.0 Dc	17.89 ± 0.5 Aab
Control (5 °C)	3.51 ± 0.0 Dc	0.65 ± 0.0 Aa	20.00 ± 0.0 BCc	18.22 ± 0.3 Aab
Gelatine (5 °C)	3.47 ± 0.0 CDb	0.67 ± 0.0 Aa	19.00 ± 0.0 Ab	17.00 ± 0.8 Aa
Gelatine + Propolis (5 °C)	3.40 ± 0.0 Aa	0.67 ± 0.0 Aa	18.00 ± 0.0 Aa	16.29 ± 1.2 Aa
**Day 10**				
Control (25 °C)	3.55 ± 0.0 CDb	0.68 ± 0.1 Aab	21.00 ± 0.0 Bd	22.24 ± 0.4 Ba
Gelatine (25 °C)	3.55 ± 0.0 Bb	0.63 ± 0.1 Aa	21.50 ± 0.0 Ce	22.73 ± 0.1 Da
Gelatine + Propolis (25 °C)	3.53 ± 0.0 Cb	0.61 ± 0.0 Aa	21.00 ± 0.0 Ed	22.13 ± 0.0 Ca
Control (5 °C)	3.51 ± 0.0 Dab	0.86 ± 0.1 Cc	18.00 ± 0.0 Aa	19.04 ± 0.1 Aba
Gelatine (5 °C)	3.46 ± 0.0 BCDa	0.80 ± 0.0 Bbc	19.50 ± 0.0 Bb	25.40 ± 9.0 Aa
Gelatine + Propolis (5 °C)	3.52 ± 0.0 BCab	0.63 ± 0.0 Aa	20.50 ± 0.0 Ec	20.56 ± 0.3 Ba
**Day 15**				
Control (25 °C)	3.70 ± 0.0 Eb	0.67 ± 0.0 Aaab	21.00 ± 0.0 Bb	19.46 ± 0.2 Aa
Gelatine (25 °C)	3.73 ± 0.0 Cb	0.60 ± 0.0 Aa	22.00 ± 0.0 Dc	20.88 ± 0.4 Ca
Gelatine + Propolis (25 °C)	3.65 ± 0.0 Db	0.73 ± 0.0 BCb	21.00 ± 0.0 Eb	19.70 ± 1.0 Ba
Control (5 °C)	3.45 ± 0.0 Ca	0.69 ± 0.0 Aab	20.00 ± 0.0 BCa	20.28 ± 0.2 BCa
Gelatine (5 °C)	3.50 ± 0.0 Da	0.72 ± 0.0 Ab	20.00 ± 0.0 Ca	19.44 ± 0.3 Aa
Gelatine + Propolis (5 °C)	3.50 ± 0.0 Ba	0.68 ± 0.0 Aab	20.00 ± 0.0 Da	19.68 ± 0.2 Ba
**Day 19**				
Control (25 °C)	3.26 ± 0.0 Aa	0.94 ± 0.0 Bc	19.00 ± 0.0 Ac	25.55 ± 0.6 Ce
Gelatine (25 °C)	3.55 ± 0.0 Bd	0.86 ± 0.0 Cbc	18.00 ± 0.0 Ab	23.84 ± 0.3 Ed
Gelatine + Propolis (25 °C)	3.44 ± 0.0 ABc	0.75 ± 0.0 Cab	17.50 ± 0.0 Aa	22.92 ± 0.4 Ccd
Control (5 °C)	3.28 ± 0.0 Aa	0.84 ± 0.0 Cabc	21.00 ± 0.0 Cd	21.39 ± 0.8 Cbc
Gelatine (5 °C)	3.36 ± 0.0 Ab	0.72 ± 0.0 ABa	19.00 ± 0.0 Ac	19.17 ± 0.3 Aa
Gelatine + Propolis (5 °C)	3.36 ± 0.0 Ab	0.81 ± 0.1 Bab	19.00 ± 0.0 Bc	20.52 ± 0.1 Bab
**Day 25**				
Control (25 °C)	-	-	-	-
Gelatine (25 °C)	-	-	-	-
Gelatine + Propolis (25 °C)	-	-	-	-
Control (5 °C)	3.29 ± 0.0 Aa	0.83 ± 0.0 BCb	22.50 ± 0.7 Da	20.49 ± 0.8 BCa
Gelatine (5 °C)	3.41 ± 0.0 ABCb	0.67 ± 0.0 Aa	22.50 ± 0.0 Da	20.51 ± 0.3 Aa
Gelatine + Propolis (5 °C)	3.40 ± 0.0 Ab	0.71 ± 0. ABa	22.00 ± 0.0 Fa	21.92 ± 1.8 Ba

* Means and standard deviations followed by the same capital letter in a column refer to the same treatment during the storage days and did not differ statistically from each other using the Tukey test at *p* > 0.05. Means and standard deviations followed by the same lowercase letter in a column refer to different treatments on the same day of storage and did not differ statistically from each other using the Tukey test at *p* > 0.05.

**Table 4:** Means, standard deviations and percentage of acceptance indexes of grape samples with and without edible toppings after 25 days of storage at refrigeration temperature (5 ± 2 °C).

Sample	Global Appearance		Color		Glow	
	Grid Average	Acceptance (%)	Grid Average	Acceptance (%)	Grid Average	Acceptance (%)
Control (5 °C)	2.56 ± 1.5	42.73	3.22 ± 1.8	40.23	2.33 ± 1.3	38.79
Gelatine (5 °C)	7.07 ± 1.4	78.59	7.36 ± 1.4	81.52	7.24 ± 1.4	80.4
Gelatine + Propolis (5 °C)	7.45 ± 1.2	82.83	7.49 ± 1.2	83.23	7.84 ± 1.0	87.07

## Data Availability

Data are contained within the article.
